# Publisher Correction: Three-dimensional atomic mapping of ligands on palladium nanoparticles by atom probe tomography

**DOI:** 10.1038/s41467-021-25765-3

**Published:** 2021-09-13

**Authors:** Kyuseon Jang, Se-Ho Kim, Hosun Jun, Chanwon Jung, Jiwon Yu, Sangheon Lee, Pyuck-Pa Choi

**Affiliations:** 1 Department of Materials Science and Engineering, Korea Advanced Institute of Science and Technology (KAIST), Daejeon, Republic of Korea; 2 Department of Chemical Engineering and Materials Science, Ewha Womans University, Seoul, Republic of Korea; 3 Graduate Program in System Health Science and Engineering, Ewha Womans University, Seoul, Republic of Korea; 4 Present Address: Department of Microstructure Physics and Alloy Design, Max-Planck-Institut für Eisenforschung GmbH, Düsseldorf, Germany

**Keywords:** Nanoparticles, Characterization and analytical techniques, Nanoparticles

Correction to: *Nature Communications* 10.1038/s41467-021-24620-9, published online 14 July 2021.

The original version of this Article contained errors in Fig. 1b and e, in which the microscopy images of the nanoparticles were inadvertently moved off-centre, and the labels representing interplanar distances and the locations of the obtained fast Fourier transform patterns were misplaced. The correct version is:  which replaces the previous incorrect version:

                  

This has been corrected in both the PDF and HTML versions of the Article.

