# Supplementary material for: Three-dimensional atomic mapping of ligands on palladium nanoparticles by atom probe tomography
Source: Nat Commun. 2021 Jul 14;12:4301. doi: 10.1038/s41467-021-24620-9 (PMC8280228; doi:10.1038/s41467-021-24620-9)
Supplement: Supplementary file 1 — Supplementary Information [file 41467_2021_24620_MOESM1_ESM.pdf]

Supplementary information for

Three-dimensional Atomic Mapping of Ligands on Palladium Nanoparticles by Atom Probe Tomography

Kyuseon Jang[1,†], Se-Ho Kim[1,2,†], Hosun Jun[1], Chanwon Jung[1], Jiwon Yu[3], Sangheon Lee[3,4✉] & Pyuck-Pa Choi[1✉]

[1]Department of Materials Science and Engineering, Korea Advanced Institute of Science and Technology (KAIST), 291 Daehak-ro, Yuseong-gu, Daejeon 34141, Republic of Korea

[2]Department of Microstructure Physics and Alloy Design, Max-Planck-Institut für Eisenforschung GmbH, Max-Planck-Straße 1, 40237, Düsseldorf, Germany

[3]Department of Chemical Engineering and Materials Science, Ewha Womans University, 52 Ewhayeodae-gil, Seodaemun-gu, Seoul 03760, Republic of Korea

[4]Graduate Program in System Health Science and Engineering, Ewha Womans University, 52 Ewhayeodae-gil, Seodaemun-gu, Seoul 03760, Republic of Korea

[†]These authors contributed equally: Kyuseon Jang, Se-Ho Kim

[✉]email: sang@ewha.ac.kr; p.choi@kaist.ac.kr

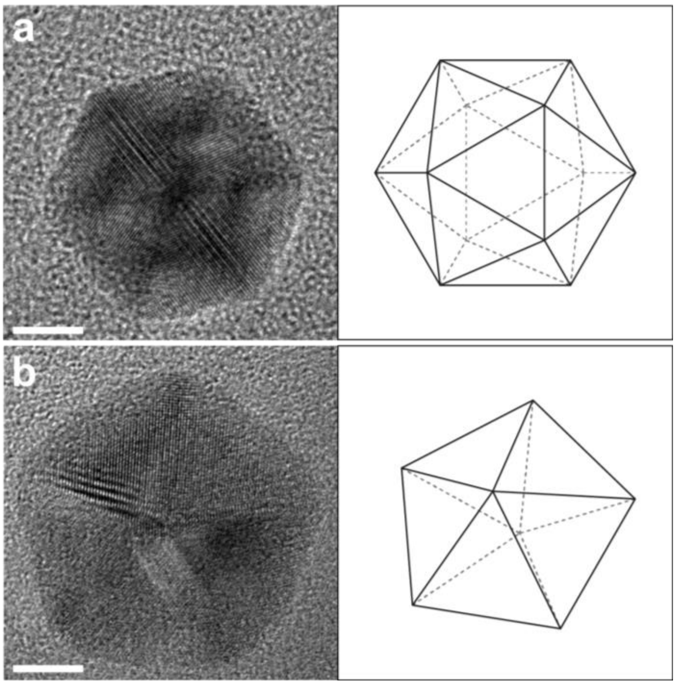

Supplementary Figure 1. TEM images and schematic drawings of NPs with multiple-twinned structures. a icosahedron and **b** decahedron. (Scale bars: 5 nm)

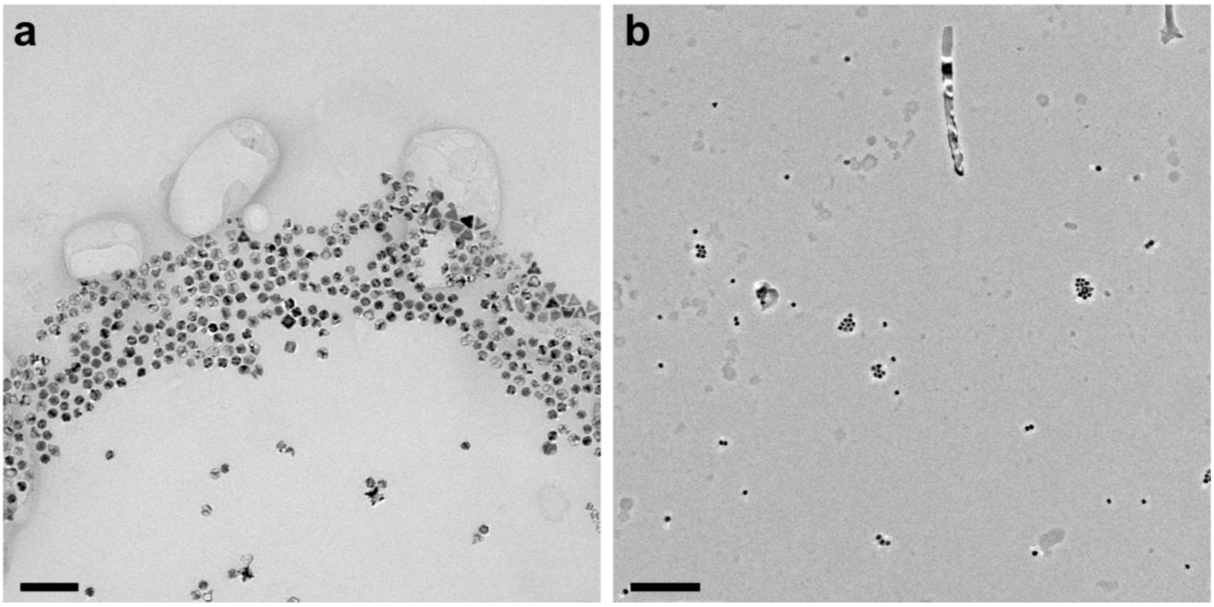

Supplementary Figure 2. TEM images of Pd NPs after storage in air for ten days. a $Pd_{(Br)}$ and **b** $Pd_{(Cl)}$ NPs. (Scale bars: 100 nm in **a** and 400 nm in **b**)

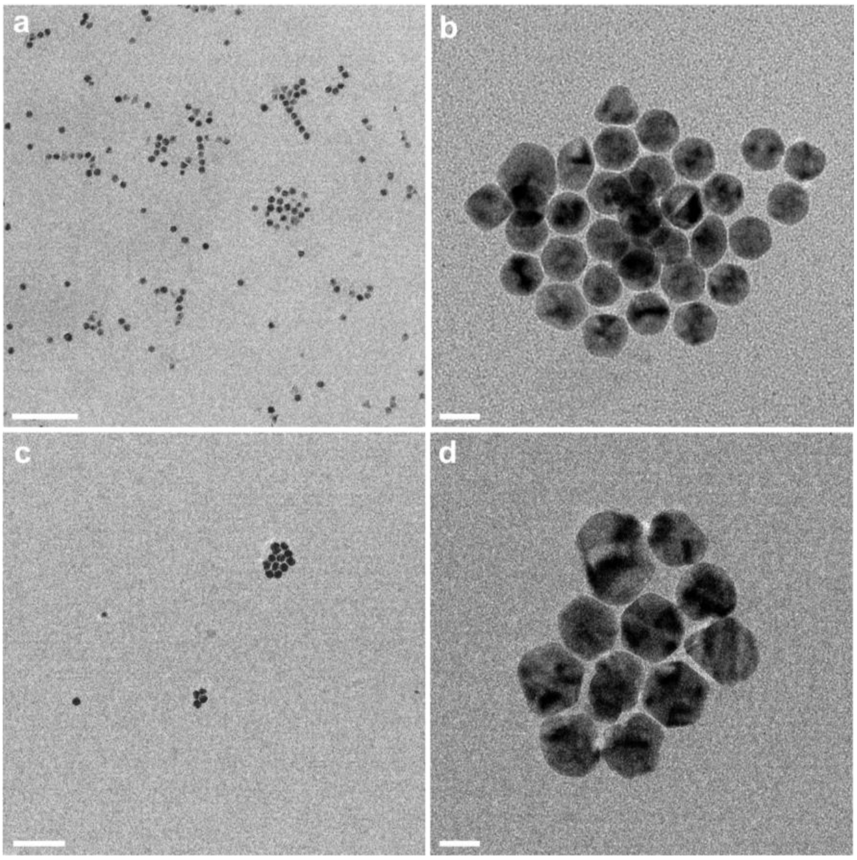

Supplementary Figure 3. TEM images of washed Pd NPs with matched halide ion concentrations left for ten days in air. a $Pd_{(Br)}$ and **b** $Pd_{(Cl)}$ NPs. (Scale bars: 200 nm in **a**, **c** and 20 nm in **b**, **d**)

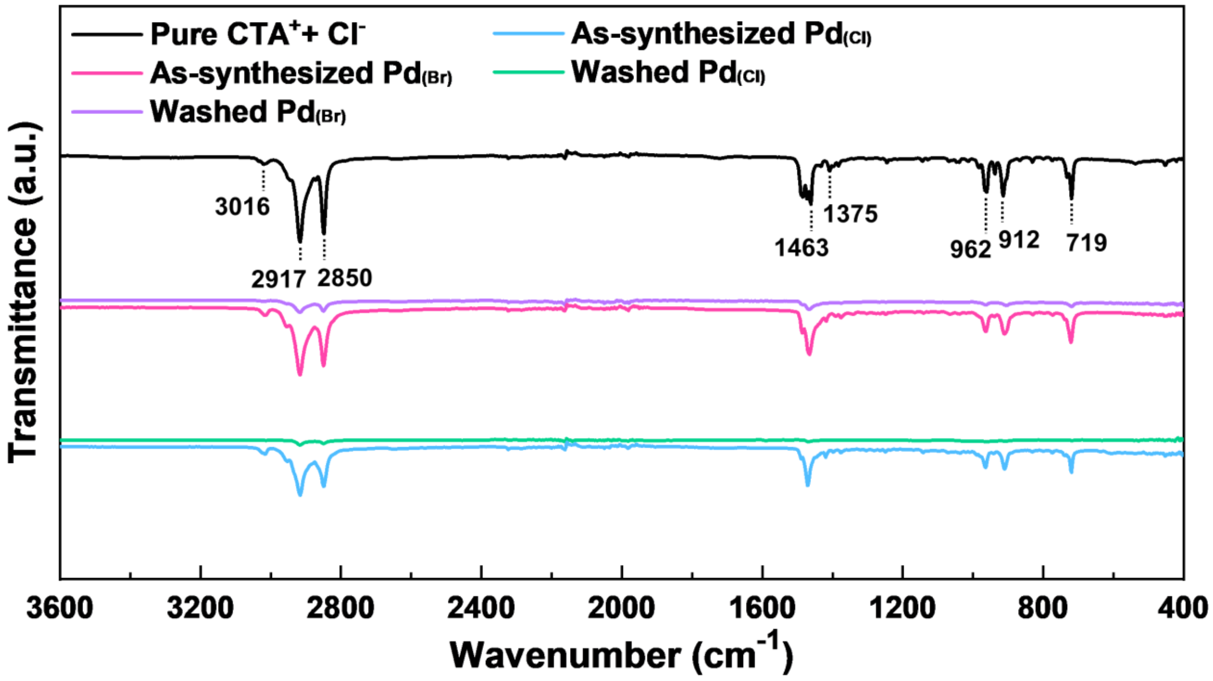

Supplementary Figure 4. Fourier-transform infrared (FT-IR) spectra. Pure cetrimonium ions (CTA^+) and Cl anions, compared with Pd NPs produced by adding Br or Cl anions ($Pd_{(Br)}$ and $Pd_{(Cl)}$, respectively), as-synthesized and after washing to remove excess CTA^+.

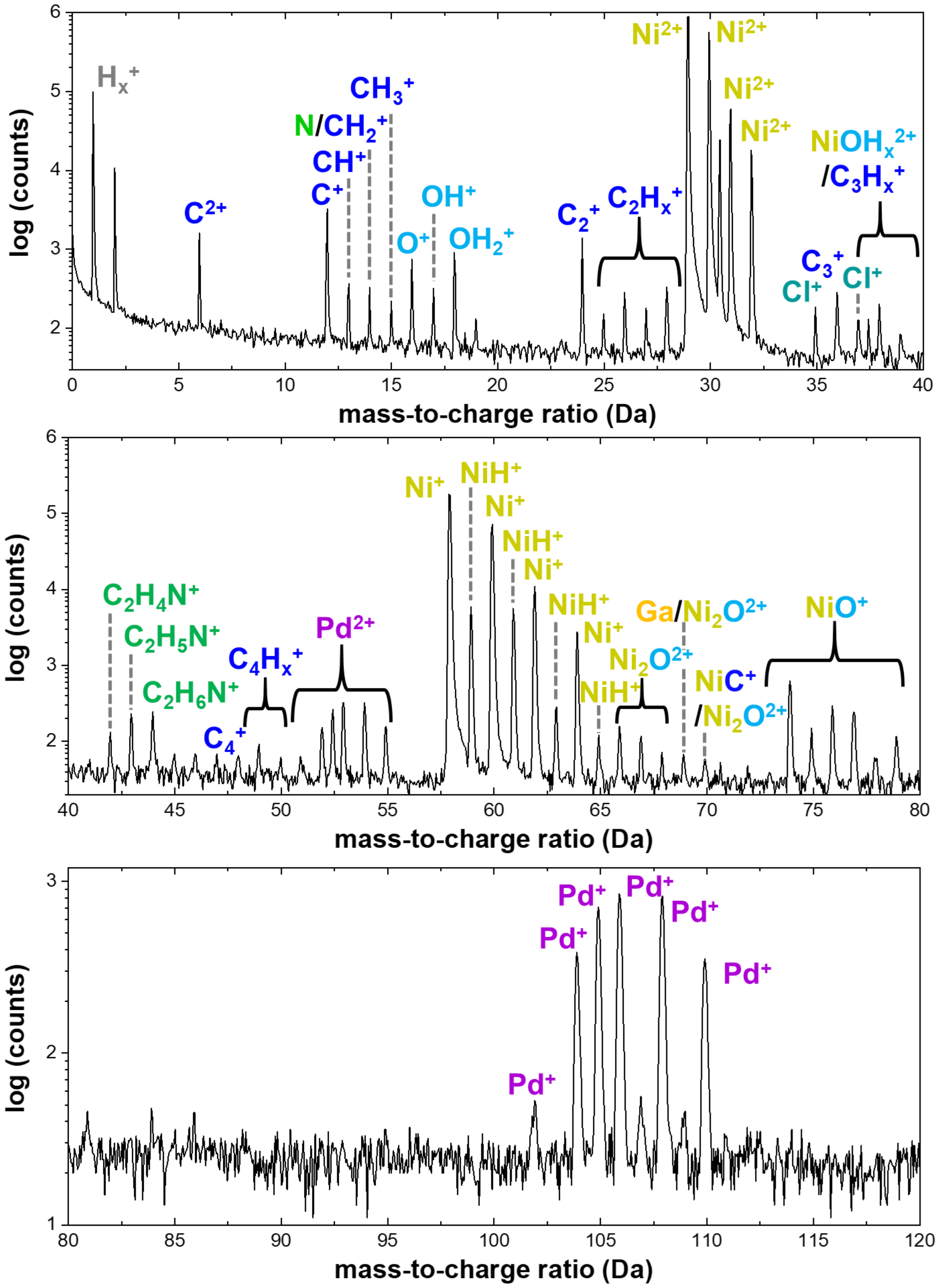

Supplementary Figure 5. APT mass spectrum acquired from $Pd_{(Br)}$ NPs embedded in Ni. The spectrum is shown for different mass-to-charge ratio ranges in the top, middle, and bottom figure.

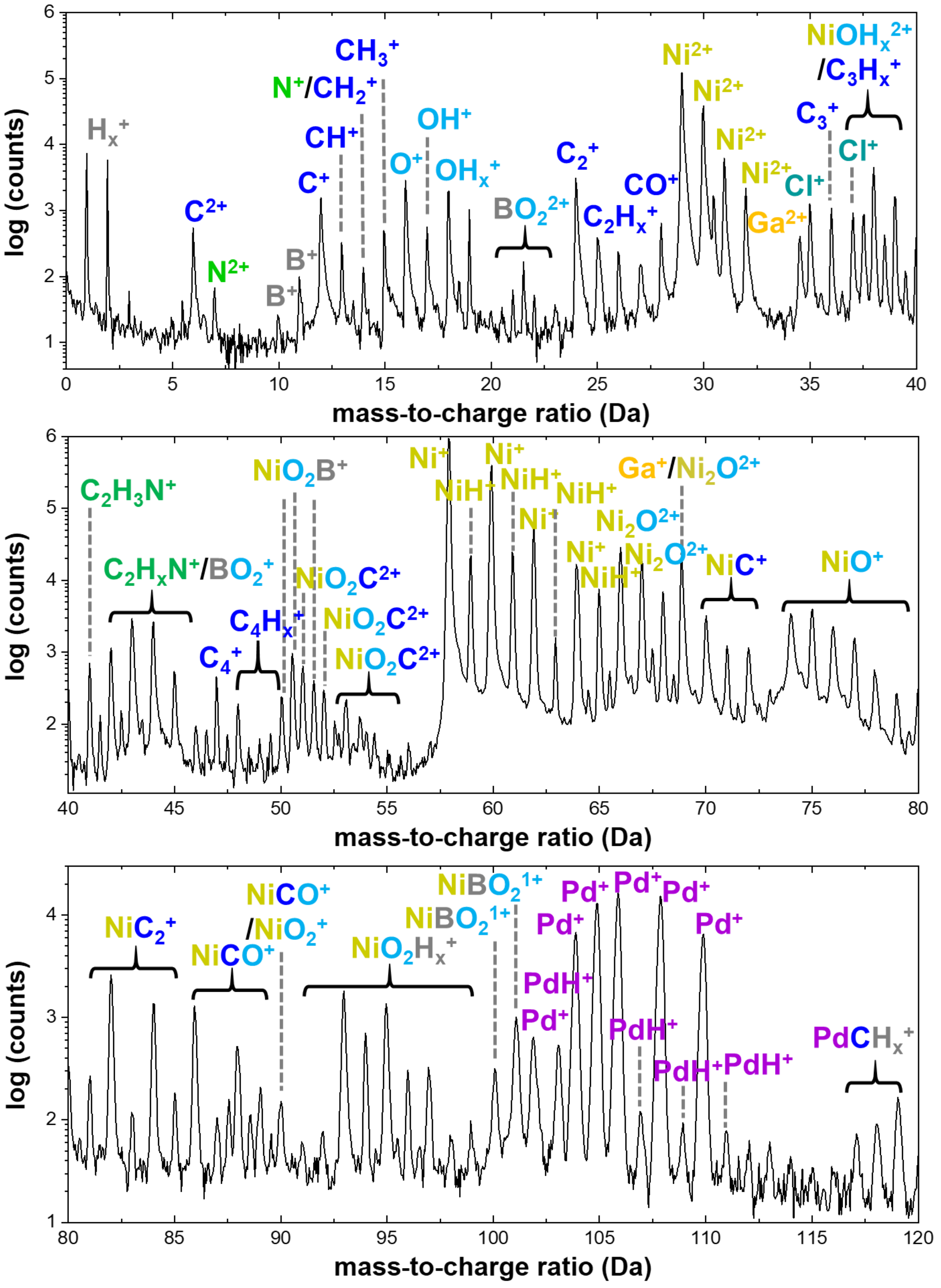

Supplementary Figure 6. APT mass spectrum acquired from $Pd_{(Cl)}$ NPs embedded in Ni. The spectrum is shown for different mass-to-charge ratio ranges in the top, middle, and bottom figure.

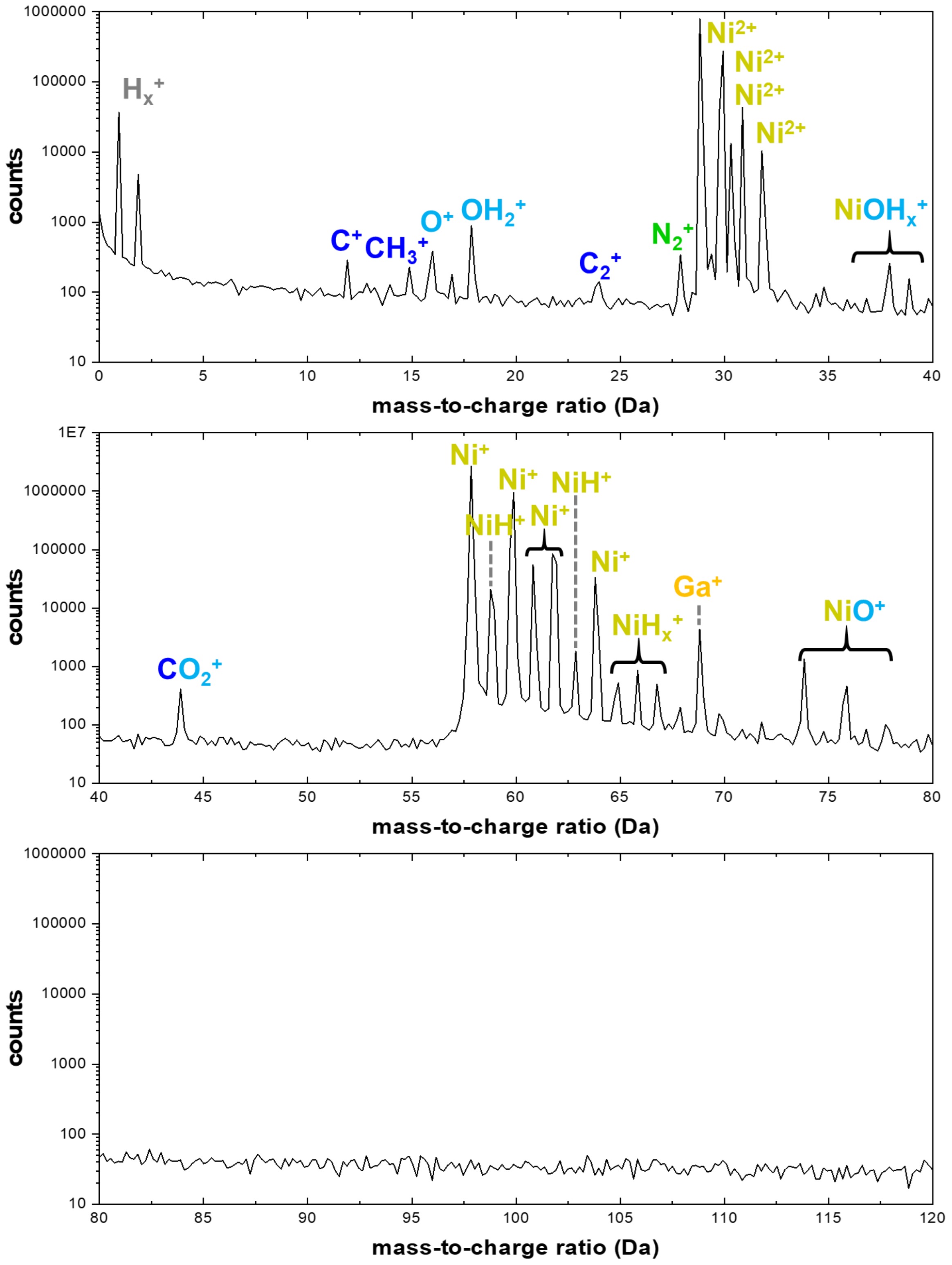

Supplementary Figure 7. APT mass spectrum acquired from an electrodeposited bare Ni specimen. The spectrum is shown for different mass-to-charge ratio ranges in the top, middle, and bottom figure.

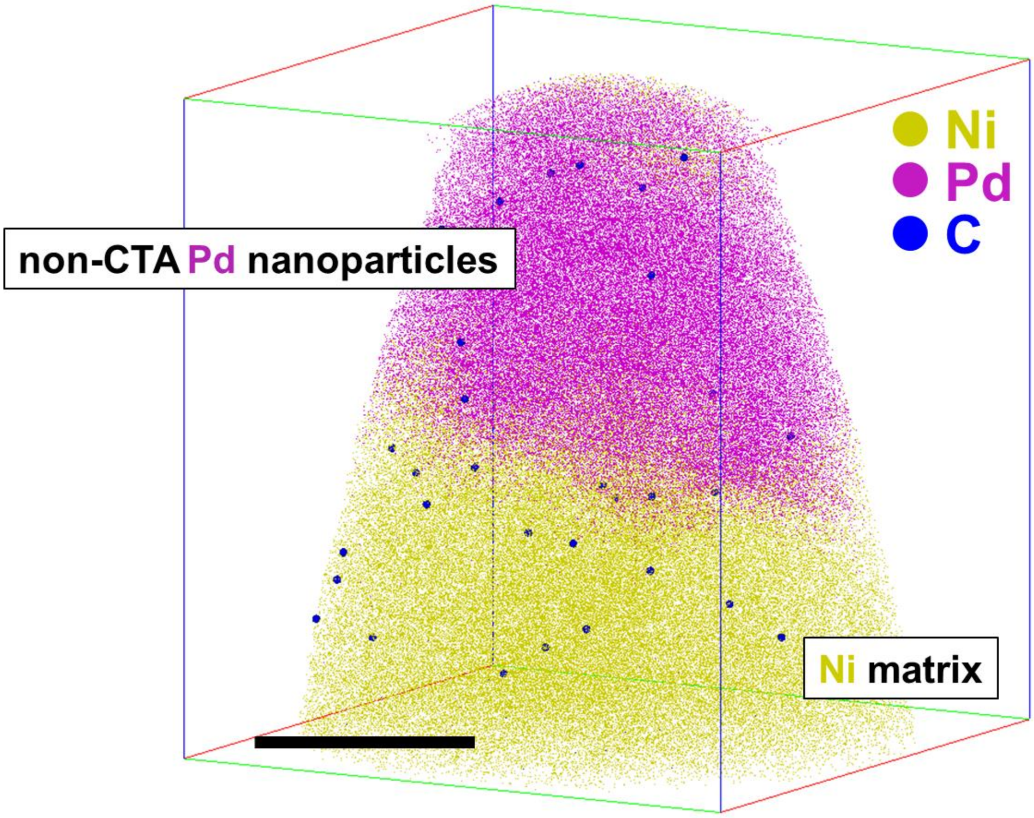

Supplementary Figure 8. APT data acquired from a Pd NP specimen prepared without CTA^{+}. Electrodeposition and FIB milling processes were identical to those applied to the $Pd_{(Br)}$ and $Pd_{(Cl)}$ specimens. (Scale bar: 10 nm)

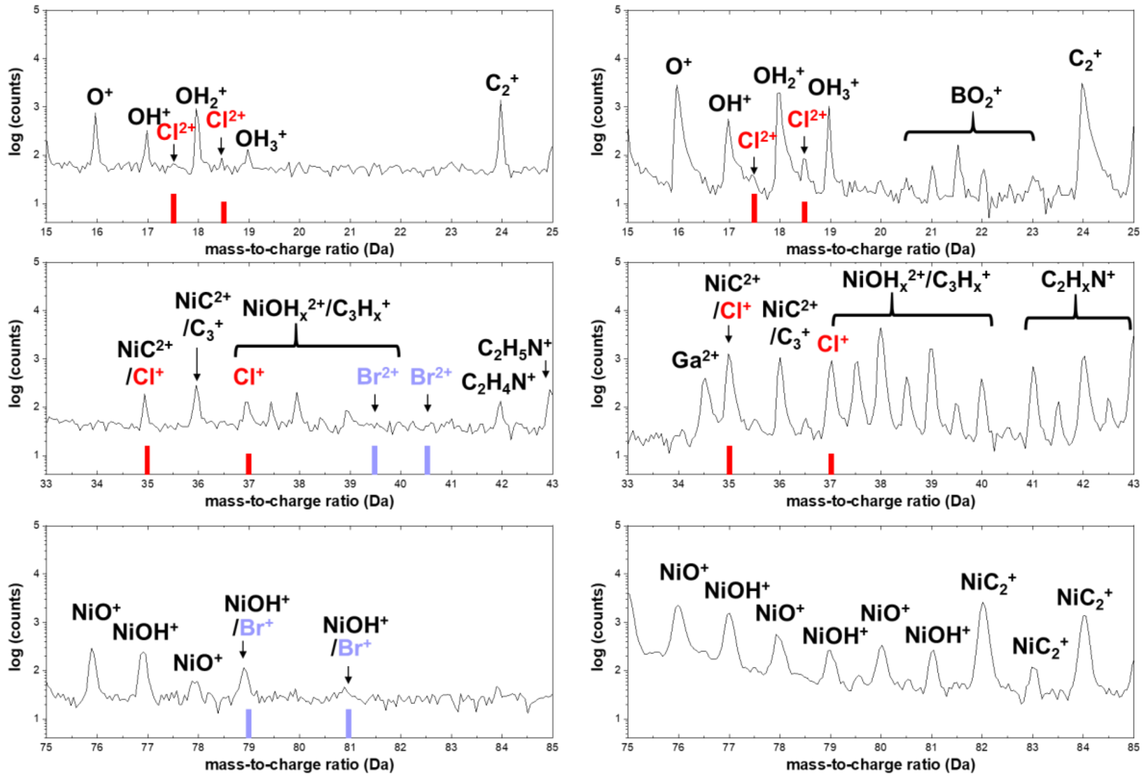

Supplementary Figure 9. Selected parts of the mass spectra of a Pd(Br) (left) and Pd(Cl) (right) specimen for closer inspection of the Br and Cl peaks. The light purple and red bars in the mass spectrum indicate the positions of the Br and Cl peaks, respectively, where their heights indicate the natural isotope ratios.

Supplementary Figure 9 shows a comparison of the mass spectra ranges of both $Pd_{(Br)}$ (left) and $Pd_{(Cl)}$ (right) specimens, where the expected positions of the Cl^{+}, Cl^{2+}, Br^{+}, and Br^{2+} peaks are marked by bars. For the $Pd_{(Br)}$ specimen, Br^{2+} peaks expected at 39.5 and 40.5 Da are below the detection limit. Slight peaks are detected at 79 and 81 Da, which can be partly assigned to Br^{+} ions. Assigning these two peaks to Br^{+} gives an upper limit for the Br concentration in the given dataset, which is 0.008 at.%. However, it is more reasonable to assign the peaks at 79 and 81 Da to $^{62}NiOH^{+}$ and $^{64}NiOH^{+}$ molecular ions, since the heights of the peaks detected in the range from 75 to 81 Da range show a good match with the natural abundance of Ni isotopes. Thus, the actual Br concentration is expected to be substantially lower than 0.008 at.%.

For both $Pd_{(Br)}$ and $Pd_{(Cl)}$ specimens, mass peaks at 35 and 37 Da were detected. Assigning these peaks to Cl^{+} yields upper limits of the Cl concentrations in the given datasets, which are 0.024 at.% for $Pd_{(Br)}$) and 0.069 at.% for $Pd_{(Cl)}$. However, the measured ratios between the peaks at 35 and 37 Da show deviations from the natural isotope ratio (1.36:1), with values of 1.08:1 for $Pd_{(Br)}$ and 1.04:1 for $Pd_{(Cl)}$. These results are indicative of the actual Cl concentrations being substantially lower than the determined upper limits.

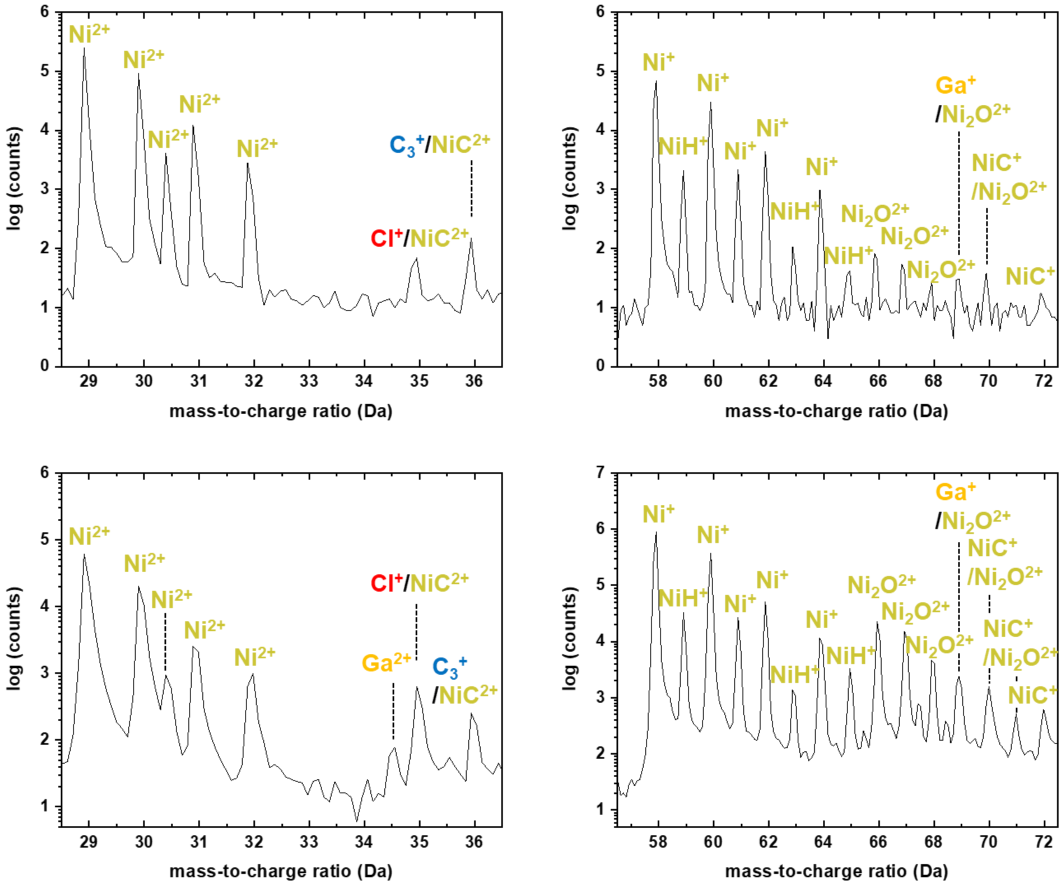

Supplementary Figure 10. Mass spectra acquired from ROIs (40×40×40 nm^3 in size) containing Pd NPs and surrounding ligands; Pd$_{(Br)}$ (top) and Pd$_{(Cl)}$ (bottom). For evaluating the upper concentration limits of the implanted Ga ions, the mass spectra are only shown in the ranges from 28.5 to 36.5 Da and from 56.5 to 72.5 Da.

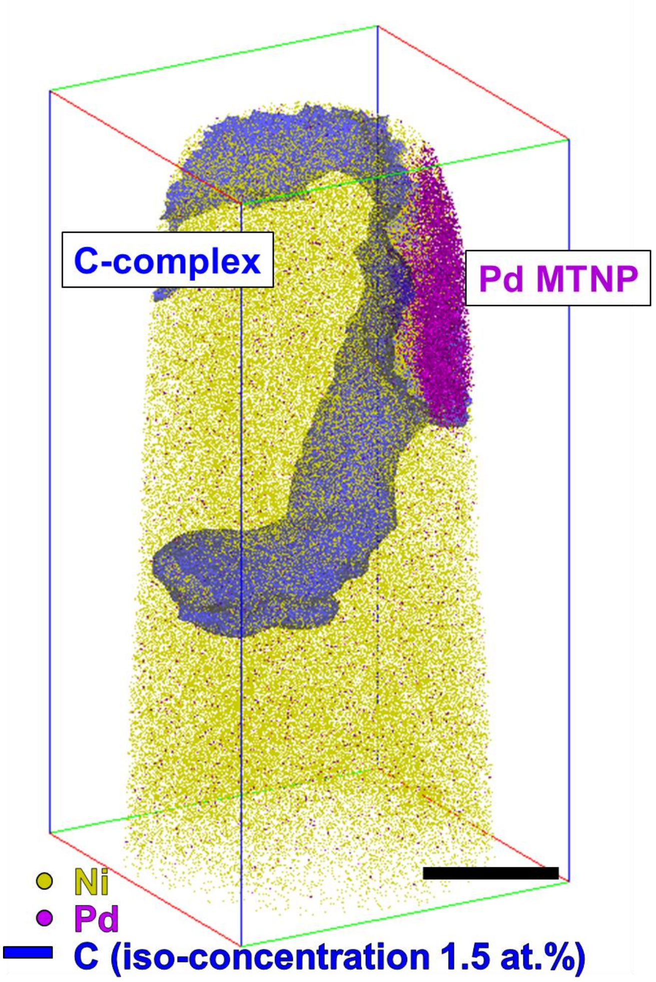

Supplementary Figure 11. Three-dimensional atom map of Pd$_{(Br)}$ NP embedded within Ni from APT. Iso-concentration surface (blue) of 1.5 at. % C highlights the locations of C-complex molecules. (Scale bar: 10 nm)

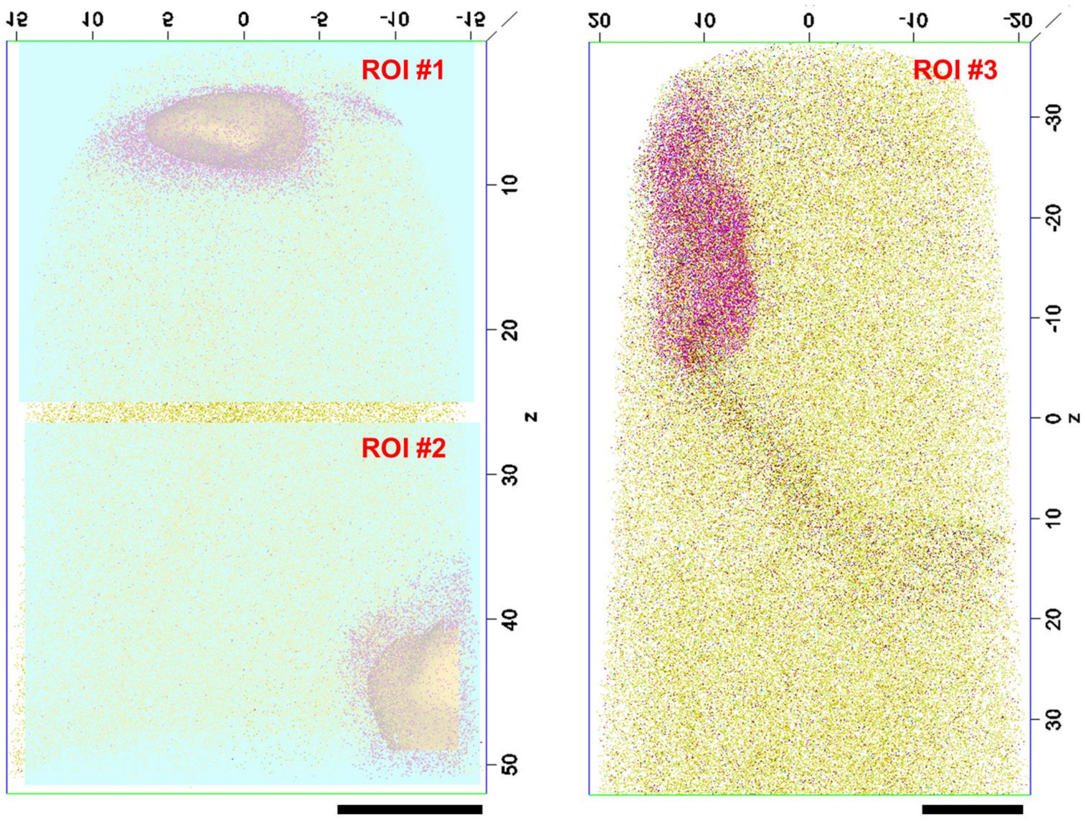

Supplementary Figure 12. Three-dimensional atom maps of $Pd_{(Br)}$ NPs and cubical ROIs from which the C:N ratios were determined. The maps correspond to the front views of the atom maps in Figure 2 (left) and Supplementary Figure 11 (right), respectively. Only the reconstructed positions of Ni (yellow), Pd (purple), and carbon (brown) atoms are shown. The map on the left was divided into two ROIs, each including a Pd nanoparticle. The map on the right was set entirely as a ROI. (Scale bars: 10 nm)

Supplementary Table 1. Number of C, N atoms and C:N ratio of each ROI shown in Supplementary Figure 12.

	C counts	N counts	C:N ratio
ROI #1	6192	344	18.0
ROI #2	1945	128	15.2
ROI #3	17221	868	19.8
Total	25358	1340	18.9

Determination of the Gibbsian interfacial excess

In order to compare the amount of the segregated CTA^+ ions on each Pd sample, the C excess was determined using the method proposed by Krakauer et al[1].

First, a cylindrical ROI was placed perpendicular across the interface between a Pd NP and Ni matrix. Next, the cumulative number of C atoms was plotted against the cumulative total number of atoms along the z-direction of the cylindrical ROI. Such a cumulative plot is also termed ladder diagram. From the ladder diagram, the total number of C atoms segregated on the Pd surface (C_{excess}) was obtained by subtracting the cumulative number of C atoms in the Ni matrix (C_{min}) from the cumulative number of C atoms at the interface (C_{max}). A linear function was fitted to the linear section of cumulative curve in the Ni matrix region to obtain the value of C_{min}. Similarly, another linear function was fitted to the linear section of cumulative curve in the Pd NP region to yield the value of C_{max}. In the cases where the linear plateau was not observed in the cumulative plot of the Pd NP region, the total number of C atoms was regarded as C_{max}, as done in Ref[2,3].

The measured C_{excess} value can be used for the calculation of the surface density of CTA^+ ions by using equation 1 in the manuscript. The calculation of the interfacial excess and the surface density of CTA^+ ions for one exemplary ROI across a $Pd_{(Br)}$ NP (ROI #5 in Supplementary Table 2) is as follows:

$$\Gamma_C = \frac{1}{A\eta} N_C^{excess} = \frac{1}{19.6\ \mathrm{nm}^2 \times 0.37}(268\ \mathrm{C\ atoms}) = 36.9\ \frac{\mathrm{C\ atoms}}{\mathrm{nm}^2} \triangleq 1.9\ \frac{\mathrm{CTA\ molecules}}{\mathrm{nm}^2}$$

The C interfacial excess is 36.9 C atoms/nm^2 for the given ROI. Since there are 19 C atoms in one CTA molecule, the surface density of the molecules is 1.9 CTA molecules/nm^2. For each $Pd_{(Br)}$ NP, four ROIs were placed across the interface between the Pd NP and the matrix. For the $Pd_{(Cl)}$ NPs, eight ROIs were randomly positioned across the interface. The determined average values of the CTA^+ surface density are 1.9 ± 0.2 and 0.7 ± 0.3 CTA^+ / nm^2 for the $Pd_{(Br)}$ and the $Pd_{(Cl)}$ NPs, respectively. While the data points are more scattered in the

case of $Pd_{(Cl)}$, the difference in the CTA^+ surface density between $Pd_{(Br)}$ and $Pd_{(Cl)}$ is clearly beyond the error range (See Supplementary Table 2 and Supplementary Figure 13).

Supplementary Table 2. Surface density values of CTA^+ ions adsorbed on $Pd_{(Br)}$ and $Pd_{(Cl)}$ specimens, as determined from ladder diagrams.

ROI	CTA^+/nm^2 $Pd_{(Br)}$	CTA^+/nm^2 $Pd_{(Cl)}$
#1	1.8	0.7
#2	1.6	0.7
#3	1.8	0.8
#4	2.2	0.2
#5	1.9	0.3
#6	1.7	0.8
#7	2.1	1.2
#8	1.7	1.1
**Average**	**1.9 ± 0.2**	**0.7 ± 0.3**

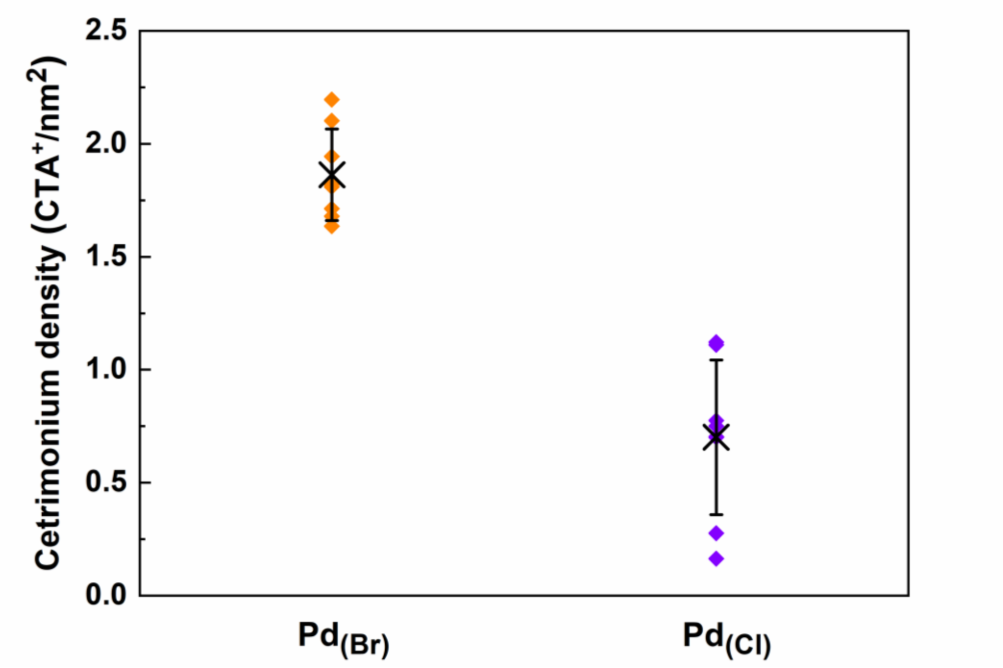

Supplementary Figure 13. Scatter plot of the surface density of CTA^+ ions on the $Pd_{(Br)}$ (orange) and $Pd_{(Cl)}$ (purple) specimens. Individual values are listed in Supplementary Table 2, where the average values are 1.9 ± 0.2 and 0.7 ± 0.3 CTA^+/nm^2 for $Pd_{(Br)}$ and $Pd_{(Cl)}$, respectively. Error bars represent a standard deviation (one sigma) and × represents an average.

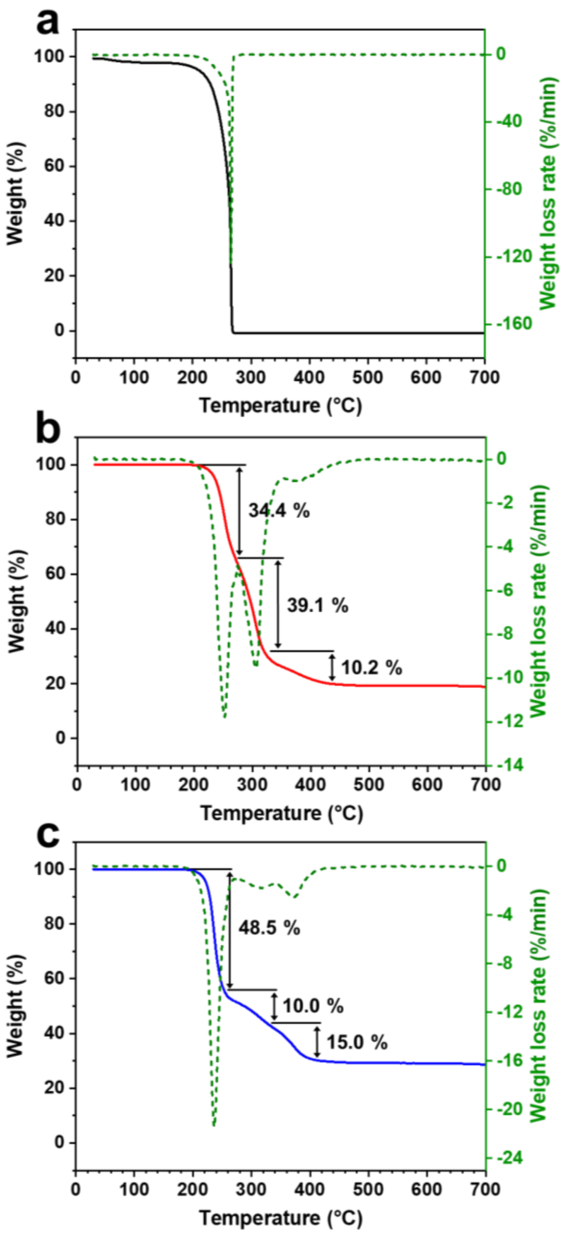

Supplementary Figure 14. TGA measurements. Weight loss curves given as wt.% vs. temperature (solid lines) and weight loss rate curves given as wt.%/min vs. temperature (dotted green lines) of **a** pure CTAC, **b** washed $Pd_{(Br)}$ NPs, **c** washed $Pd_{(Cl)}$ NPs.

Supplementary Figure 14 shows the TGA curves for pure CTAC, washed $Pd_{(Br)}$, and washed $Pd_{(Cl)}$ specimens. The TGA curve of CTAC (Supplementary Fig. 14a) exhibits only one weight loss step in the range from 200 to 260 ºC, indicating the thermal decomposition of CTAC molecules[4]. By contrast, the curves of washed $Pd_{(Br)}$ and $Pd_{(Cl)}$ NPs (Supplementary Fig. 14b, c) show three weight loss steps in the range from 200 to 275 ºC, from 275 to 345 ºC, and from 345 to 450 ºC, as seen in the weight loss rate curves. The first weight loss between 200 and 275 ºC could be due to the decomposition of unbound CTAC molecules, as seen in Supplementary Fig. 14a; the other two losses can be ascribed to the decomposition of adsorbed CTAC molecules, reflecting the bilayer structure of the CTAC molecules on the NPs[4,5]. Therefore, we consider the sum of the last two weight losses to be the residual amount of CTAC molecules on the washed Pd NPs. The $Pd_{(Br)}$ and $Pd_{(Cl)}$ NPs exhibit weight losses of CTAC of 49.3 % and 25.0 %, respectively, indicating that the amount of CTAC molecules adsorbed on the $Pd_{(Br)}$ specimen is approximately twice as high as on the $Pd_{(Cl)}$ specimen. This trend is consistent with the APT results, which revealed a larger amount of CTA^+ ions on $Pd_{(Br)}$ than on $Pd_{(Cl)}$ NPs after washing.

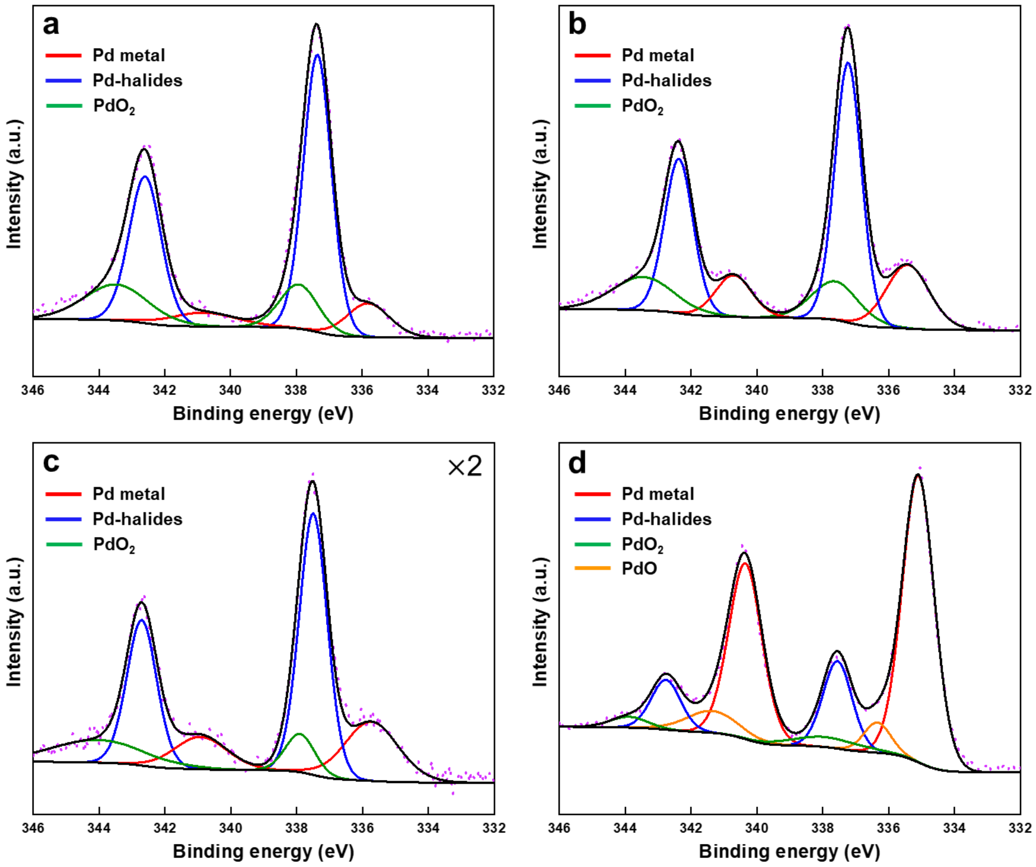

Supplementary Figure 15. XPS analyses. Pd 3d core level spectra of **a** as-synthesized $Pd_{(Br)}$ NPs, **b** washed $Pd_{(Br)}$ NPs, **c** as-synthesized $Pd_{(Cl)}$ NPs, **d** washed $Pd_{(Cl)}$ NPs.

Supplementary Figure 15 shows the Pd 3d core level spectra of as-synthesized and washed $Pd_{(Br)}$ and $Pd_{(Cl)}$ NPs. Two major peaks (fitted by red and blue lines) are observed in the spectra. The peak at 335.5 – 335.8 eV (red) is due to metallic Pd (Pd^0), while the peak at 337.3 – 337.5 eV (blue) can be ascribed to Pd atoms chemically bound to electronegative elements, such as O, Br, and Cl[6]. Since both NP specimens were synthesized in a surfactant-rich environment, the contribution of Pd oxide to the peak at 337.3 – 337.5 is low (see orange and green lines, corresponding to PdO and PdO_2 peaks, respectively[7]). Therefore, we can assign the peaks fitted by the blue solid lines to $PdBr_2$ and $PdCl_2$ for the $Pd_{(Br)}$ and $Pd_{(Cl)}$ specimens, respectively.

Both $Pd_{(Br)}$ and $Pd_{(Cl)}$ specimens exhibit a more intense Pd^0 peak and a less intense Pd–halide peak after washing. Furthermore, as shown in Supplementary Table 3, the intensity ratio between the Pd–halide complex and Pd^0 peak was always higher for $Pd_{(Br)}$ than for $Pd_{(Cl)}$. These results show that $Pd_{(Br)}$ NPs were more densely covered with ligands than $Pd_{(Cl)}$ in good agreement with the APT results.

Supplementary Table 3. Comparison of the area ratio (%) of Pd peaks for different NPs.

	As-synthesized $Pd_{(Br)}$	Washed $Pd_{(Br)}$	As-synthesized $Pd_{(Cl)}$	Washed $Pd_{(Cl)}$
**Pd metal (Pd^0)**	12.7	22.1	24.5	69.2
**Pd-halides**	66.0	58.4	59.1	16.9
**PdO**	-	-	-	8.9
**PdO_2**	21.3	19.6	16.4	5.1
**Pd-halides/Pd^0**	**5.19**	**2.64**	**2.41**	**0.24**

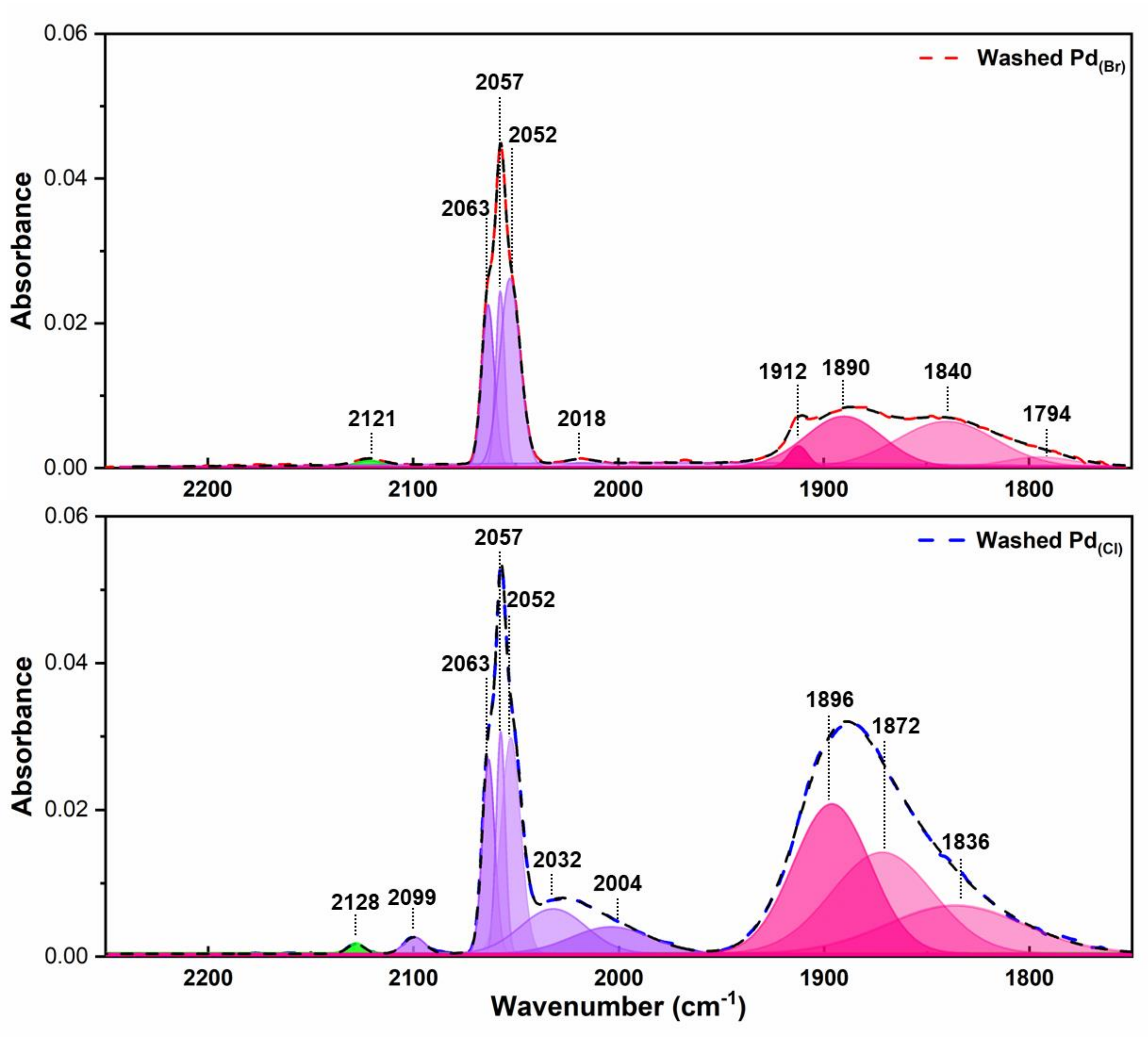

Supplementary Figure 16. Acquired (dashed lines) and deconvoluted (colored areas) CO-DRIFT spectra of washed $Pd_{(Br)}$ and $Pd_{(Cl)}$ NPs. Peaks highlighted in pink and purple can be assigned to bridged CO bonds (2000 – 1700 cm^{-1}) and linear CO bonds (2100 – 2000 cm^{-1}) on the Pd surface, respectively. Peaks at 2121 and 2128 cm^{-1} highlighted in green indicate linear CO bonds on Pd ions.

Supplementary Table 4. Wavenumber range and the assignment of each peak in the CO-DRIFT spectra of the washed $Pd_{(Br)}$ and $Pd_{(Cl)}$ NPs[8–10].

Wavenumber (cm^{-1})	Assignment	Ref
2128, 2121	Linear CO on Pd ions	[7]
2099	Linear CO on Pd {100} facets	[7]
2063 – 2052	Linear CO on Pd defects	[7]
2032, 2018, 2004	Linear CO on Pd sites / Shifted linear CO on Pd defects	[8,9]
1912 – 1836	Threefold bridged CO on Pd {111} facets	[7]
1794	Fourfold bridged CO on Pd {111} facets	[7]

Supplementary Table 4 shows the wavenumber range and the assignment of each peak shown in Supplementary Fig. 16. Compared to the CO-DRIFT spectrum of the washed $Pd_{(Cl)}$ specimen, the spectrum of the washed $Pd_{(Br)}$ specimen clearly showed lower peak intensities, especially in the 1912 – 1836 cm^{-1} region, indicating less CO adsorption on the {111} facets of $Pd_{(Br)}$ compared to $Pd_{(Cl)}$. This trend can be explained by the fact that the {111} sites of $Pd_{(Br)}$ were covered with a higher density of CTA^{+} ligands than those of $Pd_{(Cl)}$ in accord with the APT and DFT results[11].

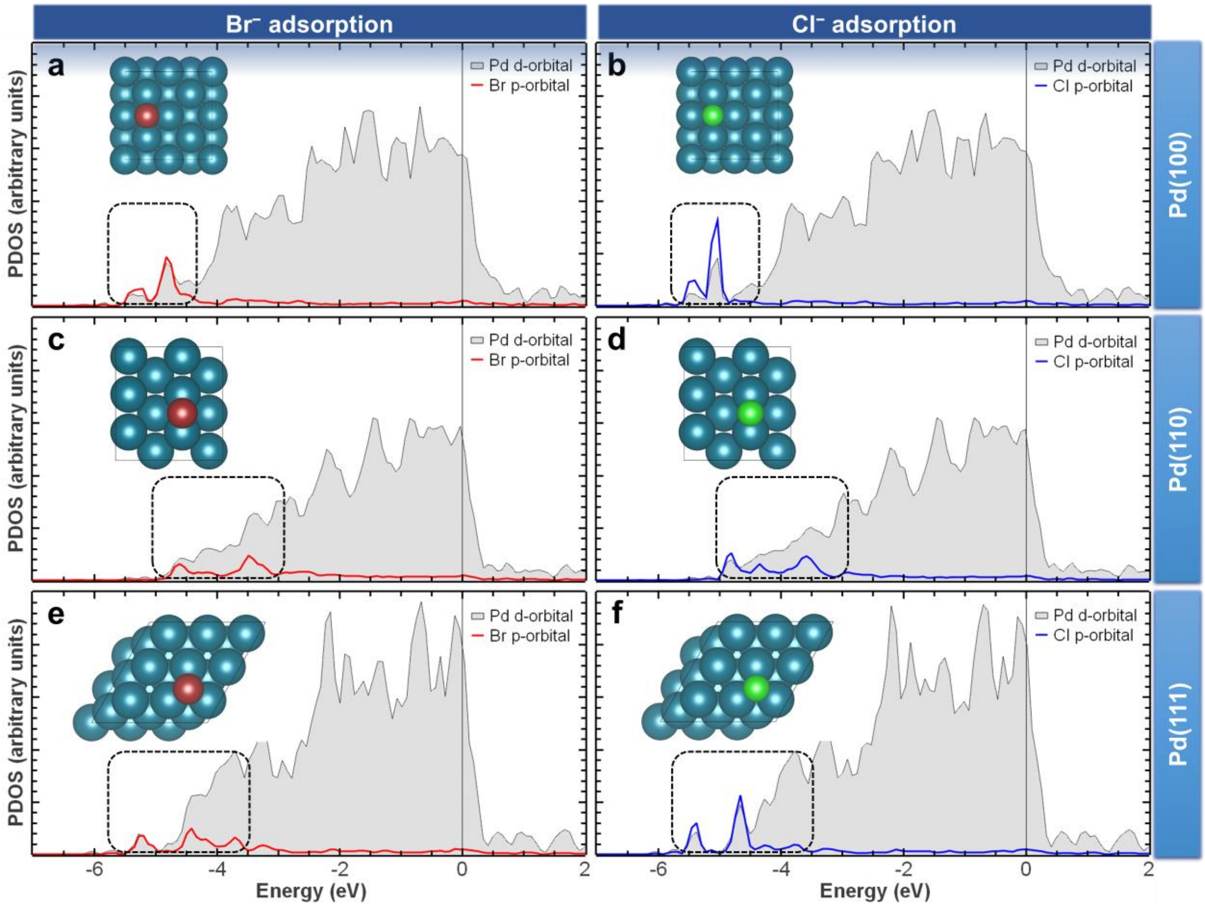

Supplementary Figure 17. Projected density of states (PDOS) for the d-orbital of surface Pd atoms and p-orbital of halide anions. **a** Pd(100)–Br^{-}, **b** Pd(100)–Cl^{-}, **c** Pd(110)–Br^{-}, **d** Pd(110)–Cl^{-}, **e** Pd(111)–Br^{-}, and **f** Pd(111)–Cl^{-}. The degree of band overlap in the dotted box indicates the degree of covalent bonding between Pd surface and halide anions. Blue, brown, and green spheres represent Pd, Br^{-}, and Cl^{-} anions, respectively.

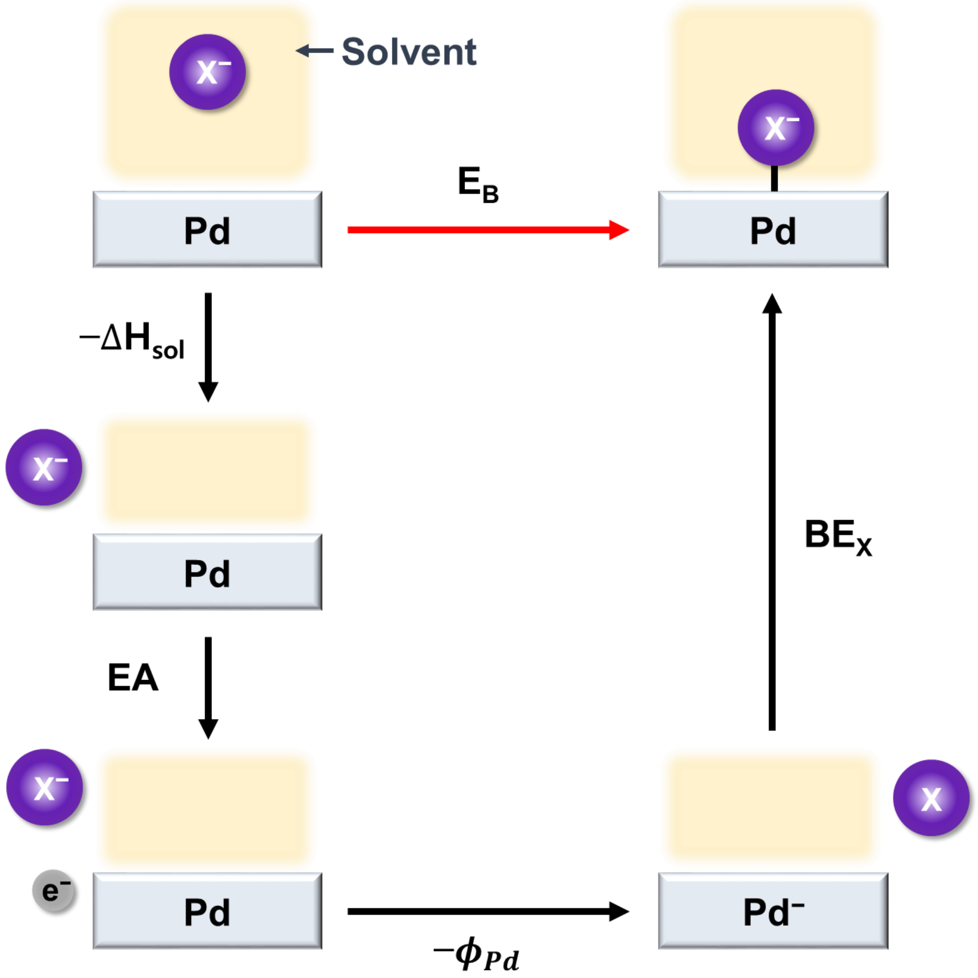

Supplementary Figure 18. Schematic illustration of the Born-Haber cycle approach employed in this work. BE_x is the binding energy for the adsorption of halogen atoms to a negatively charged Pd surface with one extra electron, ΔH_{sol}, Φ_{Pd}, and EA_x are the solvation energy of X^- in liquid water, the work function of a corresponding Pd surface, and the electron affinity of X, respectively.

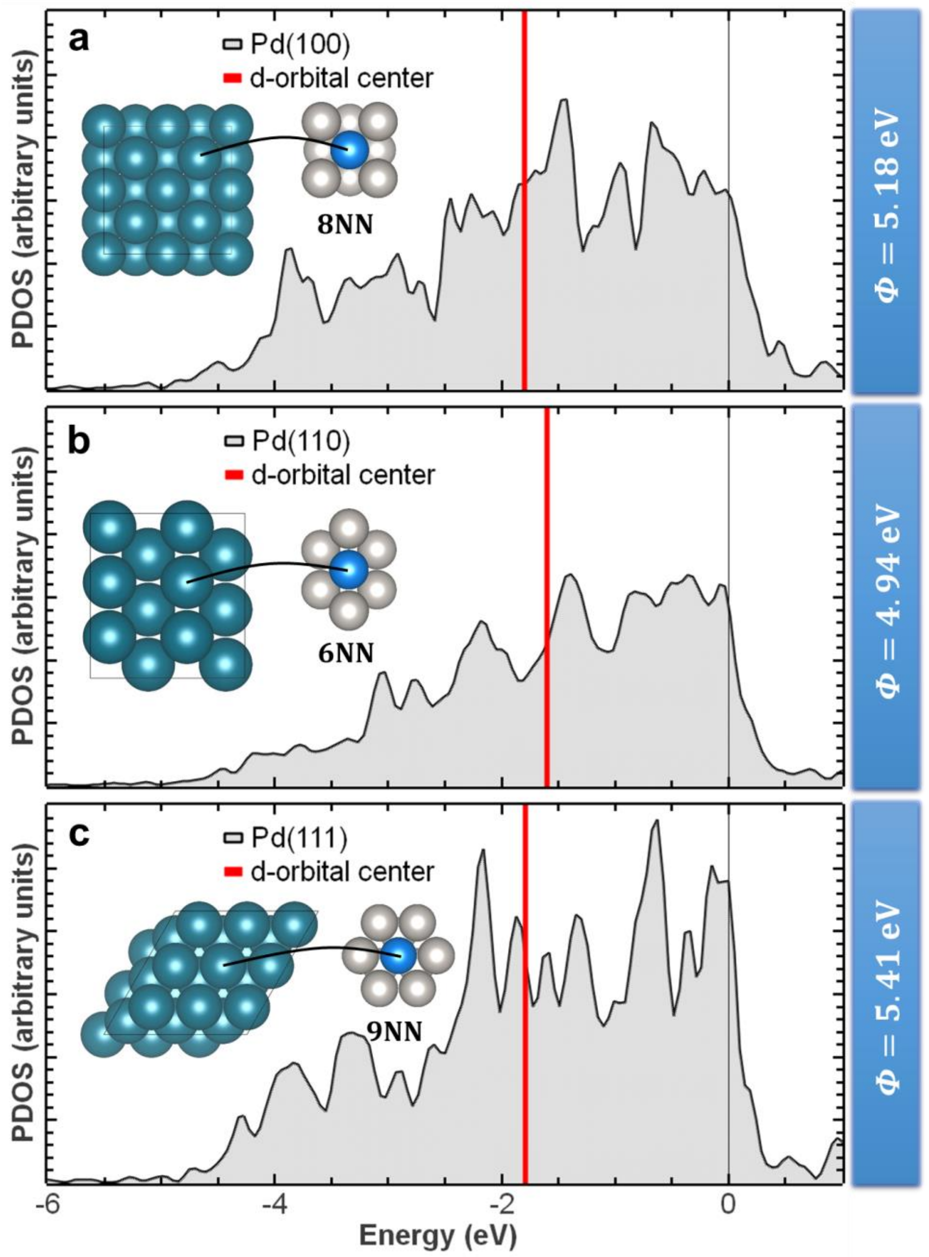

Supplementary Figure 19. d-orbital center analysis. Projected density of states (PDOS) for the d-orbital of surface Pd atoms for the bare surface of **a** Pd(100), **b** Pd(110), and **c** Pd(111). 'NN' denotes the number of nearest neighbored Pd atoms for a surface Pd atom. The calculated work functions are denoted as Φ. Blue spheres represent Pd atoms, while grey spheres represent Pd atoms neighbored to a surface Pd atom (light blue).

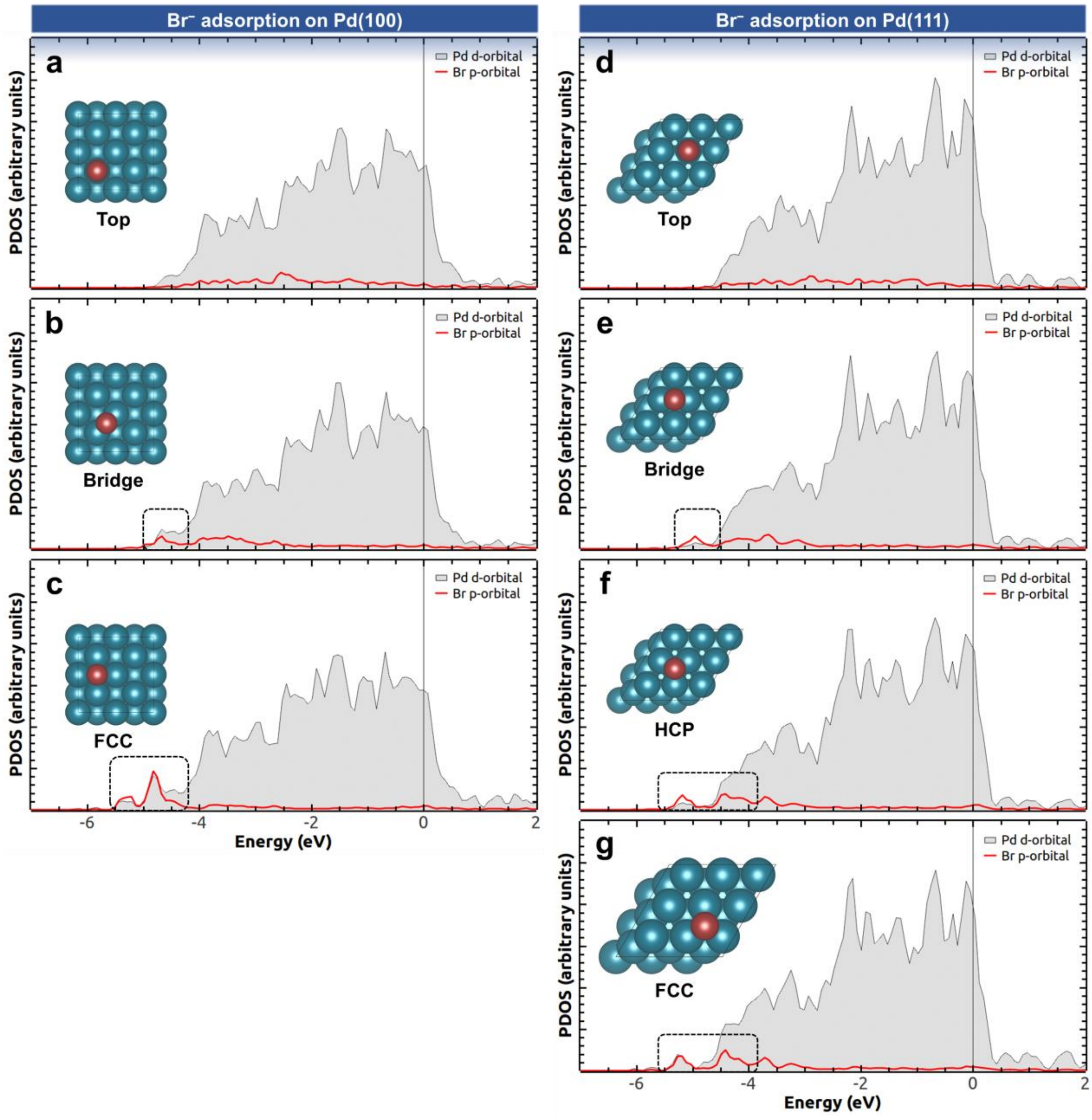

Supplementary Figure 20. Adsorption-site-specific projected density of states (PDOS) for the d-orbital of surface Pd atoms and p-orbital of Br^- anions. **a** Pd(100)–Br^- (Top), **b** Pd(100)–Br^- (Bridge), **c** Pd(100)–Br^- (Hollow), **d** Pd(111)–Br^- (Top), **e** Pd(111)–Br^- (Bridge), **f** Pd(111)–Br^- (HCP), and **g** Pd(111)–Br^- (FCC). The degree of band overlap in the dotted line indicates the degree of covalent bonding between Pd surface and halide anions. The relatively negligible band overlap at the top-site indicates that the surface Pd atoms are involved in the chemical bonding with Br^- through d_{xz} and d_{yz} orbitals instead of d_{z^2} orbitals. Blue and brown spheres represent Pd and Br^- anions, respectively.

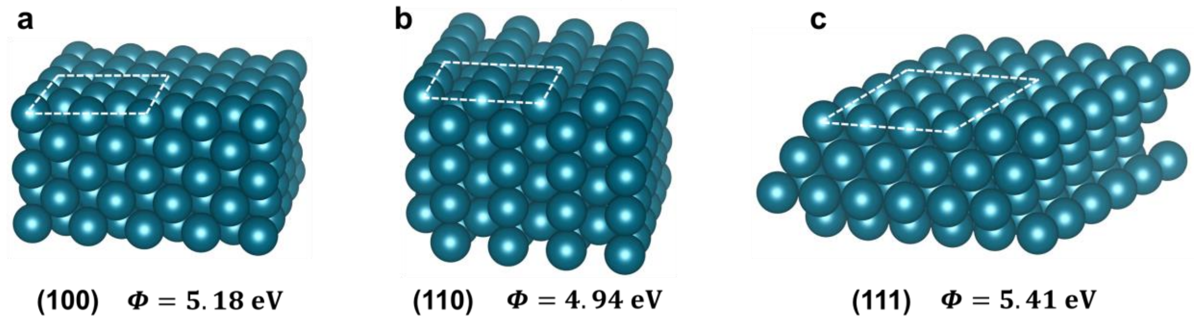

Supplementary Figure 21. Geometric structures and calculated work function (Φ_{Pd}) of the Pd surfaces. **a** (100), **b** (110), and **c** (111) surface. Dotted white lines depict supercells. The calculated work function (Φ) value is shown for each Pd surface.

Supplementary References

1. Krakauer, B. W. & Seidman, D. N. Absolute atomic-scale measurements of the Gibbsian interfacial excess of solute at internal interfaces. *Phys. Rev. B* **48**, 6724–6727 (1993).
2. Hellman, O. C. & Seidman, D. N. Measurement of the Gibbsian interfacial excess of solute at an interface of arbitrary geometry using three-dimensional atom probe microscopy. *Mater. Sci. Eng. A* **327**, 24–28 (2002).
3. Hellman, Vandenbroucke, Rusing, Isheim & Seidman. Analysis of Three-dimensional Atom-probe Data by the Proximity Histogram. *Microsc. Microanal.* **6**, 437–444 (2000).
4. Nikoobakht, B. & El-Sayed, M. A. Evidence for bilayer assembly of cationic surfactants on the surface of gold nanorods. *Langmuir* **17**, 6368–6374 (2001).
5. Sui, Z. *et al.* An improved approach for synthesis of positively charged silver nanoparticles. *Chem. Lett.* **34**, 100–101 (2005).
6. Kumar, G., Blackburn, J. R., Albridge, R. G., Moddeman, W. E. & Jones, M. M. Photoelectron Spectroscopy of Coordination Compounds. II. Palladium Complexes. *Inorg. Chem.* **11**, 296–300 (1972).
7. Kim, K. S., Gossmann, A. F. & Winograd, N. X-ray photoelectron spectroscopic studies of palladium oxides and the palladium-oxygen electrode. *Anal. Chem.* **46**, 197–200 (1974).
8. Tereshchenko, A. *et al.* Pd nanoparticle growth monitored by DRIFT spectroscopy of adsorbed CO. *Analyst* **145**, 7534–7540 (2020).
9. Zhang, L. *et al.* Efficient and durable Au alloyed Pd single-atom catalyst for the Ullmann reaction of aryl chlorides in water. *ACS Catal.* **4**, 1546–1553 (2014).

10. Zeinalipour-Yazdi, C. D., Willock, D. J., Thomas, L., Wilson, K. & Lee, A. F. CO adsorption over Pd nanoparticles: A general framework for IR simulations on nanoparticles. *Surf. Sci.* **646**, 210–220 (2016).

11. McKenna, F. M. & Anderson, J. A. Selectivity enhancement in acetylene hydrogenation over diphenyl sulphide-modified Pd/TiO2 catalysts. *J. Catal.* **281**, 231–240 (2011).
